# Bias correction and Bayesian analysis of aggregate counts in SAGE libraries

**DOI:** 10.1186/1471-2105-11-72

**Published:** 2010-02-03

**Authors:** Russell L Zaretzki, Michael A Gilchrist, William M Briggs, Artin Armagan

**Affiliations:** 1 Department of Statistics, Operations, and Management Science, The University of Tennessee, 331 Stokely Management Center, Knoxville, TN, 37996, USA; 2 Department of Ecology and Evolutionary Biology, The University of Tennessee, 569 Dabney Hall, Knoxville, TN, 37996, USA; 3 Department of Emergency Medicine, Methodist Hospital, Brooklyn, NY, USA; 4 Department of Statistical Science, Duke University, 217 Old Chemistry Bldg. Durham, NC 27708-0251, USA

## Abstract

**Background:**

Tag-based techniques, such as SAGE, are commonly used to sample the mRNA pool of an organism's transcriptome. Incomplete digestion during the tag formation process may allow for multiple tags to be generated from a given mRNA transcript. The probability of forming a tag varies with its relative location. As a result, the observed tag counts represent a biased sample of the actual transcript pool. In SAGE this bias can be avoided by ignoring all but the 3' most tag but will discard a large fraction of the observed data. Taking this bias into account should allow more of the available data to be used leading to increased statistical power.

**Results:**

Three new hierarchical models, which directly embed a model for the variation in tag formation probability, are proposed and their associated Bayesian inference algorithms are developed. These models may be applied to libraries at both the tag and aggregate level. Simulation experiments and analysis of real data are used to contrast the accuracy of the various methods. The consequences of tag formation bias are discussed in the context of testing differential expression. A description is given as to how these algorithms can be applied in that context.

**Conclusions:**

Several Bayesian inference algorithms that account for tag formation effects are compared with the DPB algorithm providing clear evidence of superior performance. The accuracy of inferences when using a particular non-informative prior is found to depend on the expression level of a given gene. The multivariate nature of the approach easily allows both univariate and joint tests of differential expression. Calculations demonstrate the potential for false positive and negative findings due to variation in tag formation probabilities across samples when testing for differential expression.

## Background

Tag-based transcriptome sequencing libraries consist of a collection of short sequences of DNA called tags along with tabulated counts of the number of times each tag is observed in a sample. These observed tag counts represent a sample from a much larger pool of mRNA tags in a tissue or organism. In the past, Serial Analysis of Gene Expression (SAGE) was the most commonly used technology for generating libraries of tag counts. SAGE libraries have been used to address a number of biological questions including: estimating transcriptome size, and estimating the density of relative expression levels. Most frequently, SAGE was used to assess differential expression across cells from different tissues or strains, or cells grown under different experimental conditions. Next generation methods such as Digital Gene Expression (DGE) tag profiling [1] now provide a more efficient method to generate tag libraries and are growing in popularity. Libraries based on DGE are used to address the same questions and provide much larger numbers of tags leading to increased statistical power. However, the close similarities between DGE and SAGE, particularly the use of restriction enzymes, lead both techniques to share the same inherent biases.

As has been repeatedly noted in both SAGE and DGE studies e.g. [1-4], the mRNA from a single gene can lead to the production of more than one type of tag within the same library. Observing multiple tags from a single gene complicates data analysis and has a number of potential causes including alternative mRNA splicing and incomplete cDNA digestion. Incomplete digestion typically results in a number of different tags being formed from the same mRNA transcript. Alternative splicing can lead to multiple mRNA isoforms within a sample. When combined with incomplete digestion, each of these isoforms may give rise to a different tag or group of tags.

While it is intuitively sensible to analyze the prevalence of an mRNA by aggregating multiple tags derived from it, such tags are often analyzed separately [5]. At best, this approach diminishes the statistical power that is available when testing differences in mRNA abundances and reduces the accuracy of inferences based on asymptotic statistical methods (chi-square tests). More seriously, such comparisons can result in dramatic biases leading to erroneous conclusions in testing differential expression due to varying levels of incomplete digestion across libraries.

The current work extends an earlier analysis [6] based on SAGE libraries generated using the yeast *Saccharomyces cerevisiae *[7]. Alternative splicing is generally rare in microorganisms such as *S. cerevisiae *so that the tag multiplicity observed in these libraries is primarily attributed to incomplete cDNA digestion. The first objective of the current work is to provide a methodology that allows multiple tags, which arise due to incomplete digestion, to be combined and used to infer expression levels of the underlying mRNA transcripts. Aggregation is complicated by the fact that certain tags are more likely to form than others due to processing methods [4,6]. Unless the tag formation probabilities are accounted for, inferences based on aggregate tag counts may be highly biased.

Previously, we have addressed the problem of estimation bias by directly modeling the variation in tag formation and deriving bias corrected maximum likelihood and posterior mode estimators of the composition of the mRNA population [6]. However, a number of assumptions and difficulties limit the application of these techniques. We address these in the present study. Three new hierarchical models for SAGE data are introduced that directly correct for the bias by embedding a model of tag formation within the probability distribution describing the data. Corresponding Gibbs sampling algorithms suitable for model fitting and inference are also derived. These methods are computationally efficient and allow a more general class of prior distributions than earlier analytic approaches. The results are also applicable for both aggregate and standard tag counts. We use simulation studies and analysis of publicly available data from *S. cerevisiae *[7] in order to contrast the accuracy of our sampling methods and analyze the effects of priors. The approach we present should serve as a building block for a more comprehensive method that takes into account the effects of both incomplete digestion and alternative splicing.

A second major objective of the current work is to explore the general effects of tag formation bias on differential expression. We carefully demonstrate that, in the absence of proper adjustments, variation in tag formation probability across experiments can lead to both false positive and false negative conclusions regarding the differential expression of a gene. A brief description of the implementation of proposed algorithms in differential expression analysis is also provided. While the approaches presented here require more work in processing and preparation than studies that focus on individual tags, they may offer a significant advantage with respect to statistical power in studies of differential expression.

A number of statistical methodologies have been developed to analyze SAGE libraries. For studies that focus on relative expression levels, individual tags are viewed as possible outcomes from a multinomial sampling model [7,8]. [9] offered an improvement on this by directly applying a Bayesian multinomial-mixture Dirichlet model to the observed vector of tag counts which improves accuracy in estimation of tag frequency. Marginal analysis may be more useful in studies of transcriptome size and was developed by [10] and [11,12]. Statistical methods to assess differential expression across a number of samples were discussed by [13-16]. These analyses typically test the differential expression of each tag across libraries independently; see [14].

### The SAGE Methodology

The goal of a SAGE experiment [8] is to sample the mRNA population within a group of cells. Broadly speaking, the SAGE method generates a set of short sequence-based cDNA tags from the mRNA population. Initially, a pool of mRNA is extracted from a group of cells. The unstable single stranded mRNA is reverse transcribed into a double stranded DNA copy (cDNA) using a modified primer that allows the cDNA to be bound to a streptavidin bead. Using restriction enzymes, the bead-bound portion of cDNA is cut into small 'tags' that are concatenated together into longer cDNA molecules referred to as concatemers. These concatemers are amplified using PCR and then sequenced. A cleavage motif of the anchoring enzyme allows the start and stop points of tags to be identified within a concatemer. Each time a tag from a specific gene is observed in the sequence data, it contributes a count to the library. The DGE technology follows a similar procedure in the first several steps. However, tags are neither concatenated nor cloned but sequenced immediately.

In both technologies the restriction enzymes used to create the tags cut at very specific sites within the cDNA. For example, the restriction enzyme used by [8] cleaves only at the four nucleotide motif CATG. Thus, tags can only be created at specific points within a cDNA. Because the site where the cDNA is cut serves as an 'anchor' between tags, the restriction enzyme used is often referred to as the anchoring enzyme (AE). We refer to the specific points in the cDNA cleaved by the anchoring enzyme as AE sites.

**Figure 1:**
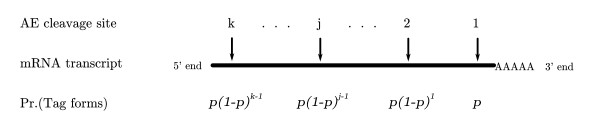
**Tag formation model**. Plot showing cDNA cleavage sites for SAGE with associated probabilities of tag formation. Adopted from [[6], Figure 1].

While some genes will have no AE sites, many genes have multiple sites at which the AE could cleave the cDNA. Given that the AE is expected to act in a site-independent manner, a single cDNA molecule can be cut by the AE in multiple places. However, because only the fragment of cDNA that is attached to a streptavidin bead is retained during the experimental process, the site closest to the bead (i.e. the 3' most site) that is actually cleaved is the only site that can lead to an observable tag (see Figure 1). If the AE worked with 100% efficiency, then each mRNA could only lead to one observable tag. However, the cutting efficacy of the AE is always less than 100%, sometimes referred to as incomplete digestion, and as a result multiple tags may be generated from the cDNA of a single gene. The critical point is that differences in the number of AE sites between genes result in different probabilities of tag formation. The most extreme cases are genes whose mRNA transcript lacks any AE site. Such genes are impossible to observe using the SAGE methodology. For genes with AE sites, differences in digestion efficiencies lead to differences in tag formation probabilities between experiments.

A further complication is that many of these sites lead to non-unique or 'ambiguous' tags that cannot be readily assigned to a particular gene. Ambiguous tags are 'uninformative' using current technologies; tag-to-gene mapping is discussed at length in [17]. The tags generated in the experiment analyzed below were 14 bp long leading to a number of ambiguous tags. Experimental advances, for example, 'SuperSAGE' techniques can lead to tags up to 26 bp long [18,19] significantly diminishing this problem.

### Modeling Tag Formation Probabilities

The previous discussion leads us to postulate that genes with larger numbers of AE sites have a increased probability of tag formation and further that there is large variation in the probability of tag formation within a gene. Below we present a quantitative model for tag formation probabilities within a gene that is key to later bias correction methods.

Because only cDNA that is attached to a streptavidin bead is retained during the experimental process, tags are created from the 3' most AE site that is actually cleaved (Figure 1). Let *k*_*i *_be the number of restriction enzyme sites which may be cleaved by the AE on mRNA generated from gene *i*, let *p *be the probability the AE will cleave a site, and assume that cleavage probability is independent between sites and does not vary between genes. If we label sites 1 through *k*_*i *_starting at the 3' most site (i.e. the site closest to the bead; see Figure 1), the probability of generating a tag through AE cleavage at site *j *∈ {1, ..., *k*_*i*_} follows a geometric distribution, (1 - *p*)^*j*-1^*p*. This corresponds to the probability of no cleaving in sites 1 to *j - *1, and a cleaving at site *j*. Note that this probability is independent of what happens at the AE sites 5' from the *j*th site. The fact that the expected distribution of tags varies with AE cleavage probability *p *can be used to estimate *p *from the library of tag counts; see [6].

It follows that the probability of generating any of the possible tags from the *i*^th ^category of mRNA follows a geometric distribution(1)

where the sum is over all non-ambiguous tagging sites. We refer to this quantity as the *tag formation probability*.

For purposes of our analysis, we will assume that, because each of the thousands of genes provides an independent estimate, the value of *p *is known to a very high precision. The variation in the tag formation probability between genes stems from variation in the number of AE sites as well as the number of ambiguous tagging sites. Given *p*, the only uncertainty in the estimate of *ϕ*_*i *_results from ambiguous tags; this problem may be partially eliminated through techniques that generate longer tags; see [19]. Hence, for methodological purposes we reasonably assume the *ϕ*_*i *_to be *known *constants (see [6] for a more details on these calculations). As a result, the tag pool represents a biased sample of the mRNA population of interest being a function of both the *known *distribution of tag formation probabilities across the genome, and the distribution of these genes within the mRNA population itself.

Traditionally, a gene's frequency in the tag pool was assumed to be an unbiased estimate of its frequency in the mRNA population, the population of interest. This equivalence only holds when all genes have the same tag formation probability, a condition that is never met. Consequently, genes with greater than average tag formation probabilities will be over-represented while those with lower than the average probability will be under-represented. Equation (1) allows us to accurately estimate this sampling bias and correct the inferences made from tag libraries.

Before the tag counts are considered, we assume that the data is processed to retain only informative tags, i.e. all ambiguous or orphan tags are removed, as is standard practice. Let the vector **t **= (*t*_1_, ..., *t*_*l*_) represent the aggregated observed tag counts for individual genes. Here *l *is the number of genes that contain at least one unambiguous AE site and *t*_*i *_is the total number of observed tags that can uniquely be attributed to an AE site in gene *i*. (Note that standard dis-aggregated counts are simply a special case of the aggregated counts given above with *ϕ*_*i *_representing a single element within the summation term in Eq. (1).) It is natural to view this vector as a sample from a multinomial distribution with *l *categories, **t **~*Multi *(*t*_*tot*_, ***θ***) where ***θ ***= (*θ*_1_, ..., *θ*_*l*_), *θ*_*i *_represents the frequency of tags from gene *i *in the tag pool, and  is the total count of all observed tags. Because the variation in tag formation probabilities *ϕ*_*i *_distorts the abundances of observed tags, it is clear that *θ*_*i *_represents a biased estimate of the true proportion of mRNA from gene *i *in the overall pool. Unfortunately, this true proportion of mRNA, denoted *m*_*i*_, is clearly the quantity of interest. [6] relates the biased proportion *θ*_*i *_to *m*_*i *_with the following simple expression:(2)

where *ϕ*_*i *_is the *known *tag formation probability based on Eq. (1) and the proportions *m*_*i *_are positive and sum to one. The bias corrected likelihood model is then,(3)

Direct maximization of the likelihood with respect to the parameters *m*_*i *_is straightforward due to the fact that the likelihood function is maximized at the same location irrespective of the way the model is parameterized. Consider the observed sample proportion  = *t*_*i*_/*t*_*tot*_. The fact that the *m*_*i *_are positive and must sum to one along with Eq. (2) force the maximum likelihood estimates of  to satisfy the following equality,(4)

While computation of the maximum likelihood estimates (MLE's) is relatively straightforward, constructing confidence intervals for **m **is difficult. The existence of the normalization term ∑_*j*_*m*_*j*_*ϕ*_*j *_in the denominator of the left hand side of Eq. (4) makes computation of Fisher's information matrix taxing, particularly for the large dimensional vectors encountered when working with SAGE data (i.e. *l *is of the order 10^3 ^to 10^4^). In addition, because the number of categories is generally within an order of magnitude of, or possibly even greater than, the number of tags sampled, most categories have either zero or only a few observations. These relatively small counts call into question inferences such as chi-squared tests which are based on asymptotic approximations.

As an alternative, we consider a Bayesian approach to the problem. The constraints lead naturally to the assumption of a Dirichlet prior on **m**,

where *α*_*i *_is the parameter describing any prior information we have on the value of *m*_*i*_. Combining this prior distribution with the likelihood function given in Eq. (3) leads to a posterior distribution proportional to the product(5)

[6] introduces methods for the direct maximization of this quantity and discusses the choice of a prior and its consequences on the marginal inferences for individual values of *m*_*i*_. However, a number of difficulties severely limit the range of prior parameters that can be used; i.e. those priors with appreciable weight relative to the observed sample sizes. Given the bias in the observed data, the Gibbs sampling based approaches for inference based on the posterior of the mRNA proportions introduced here are more flexible, and require fewer assumptions than analytic techniques. The methods are also highly computationally efficient. While the assumption of independent cleaving resulted in a geometric model, it is important to note that alternative models of tag formation can be substituted into the Bayesian algorithms without making any further adjustments to the model.

### Shrinkage Estimation in Multivariate Models

Joint estimators of the relative proportions of individual tags in SAGE experiments based on posterior means from a conjugate Dirichlet-Multinomial model were explored by [9] and have similar properties to the methods proposed here. In the case of *flat *priors (i.e. *α*_*i *_= 1 for all *i*), they note that while the posterior mean provide improved average accuracy with respect to sums of squared errors across all categories, estimates of categories where large counts are observed were shrunk excessively in order to boost the estimated probabilities of cells with few or no counts. Due to the massive number of categories and the extreme imbalance in observed frequencies (e.g. more than half of the categories have zero counts in our data), the estimates for frequently observed genes tend to consistently underestimate the true proportions while the reverse is true of genes with very low expression proportions. This observation highlights the main weakness of *shrinkage *estimators such as the posterior mean. While they perform better on average across categories, they may perform poorly on particular categories that may be of primary interest. [9] addresses this issue by proposing a mixture of Dirichlet-Multinomial's but does not discuss the issue of sampling bias. Note that after aggregation, the large reduction in the number of categories significantly reduces the side effects of shrinkage leading to improved inferences. These observations will be useful in analyzing the bias corrected models proposed below.

## Results and Discussion

### Hierarchical Models and Gibbs Sampling

Three hierarchical approaches are proposed to model aggregate tag counts while taking into account the tag formation probability. The Dirichlet-Poisson-Binomial model (DPB) extends the method of [10], who modeled tag counts as independent random variables from a Poisson-Gamma mixture to a multivariate setting that models the joint distribution of tags. The Dirichlet-Multinomial-Binomial model (DMB) views the unobserved true counts of mRNA in the population as being derived from a multinomial distribution. The missing-data model (MD) assumes that if the number of mRNA transcripts that were not converted into tags due to incomplete digestion by the anchoring enzyme, denoted *r*, were known then the data could be modeled as a standard Dirichlet-Multinomial model. By proposing a Poisson distribution for *r*, posterior inferences can be made. Detailed descriptions for these algorithms are given in the Methods section. Gibbs samplers based on these algorithms all produce posterior samples of **m**, the vector of relative proportions of each gene in the mRNA population. In addition, DPB produces samples representing *N*, the hypothetical total number of mRNA transcripts in the population while MD produces posterior samples of *r*, the number of unconverted transcripts. These quantities may be relevant in studies of the number of unique transcripts.

A Dirchlet prior distribution was assumed for the proportion vector **m **in all three algorithms. Two parameter vectors  = (*α*_1_, ..., *α*_*l*_) were tested. The *flat *prior sets *α*_*i *_= 1 for all *i *while the *tub *prior sets *α*_*i *_= 1/*l*. The flat prior effectively assumes that a hypothetical previous experiment produced 1 tag for each category. As the name suggests, the flat prior represents a uniform density over the parameter space. The *tub *prior assumes that the prior information is equivalent to that of a single tag. The density of this prior takes a bowl or tub shape. The preponderance of mass along the edges forces the estimates of *m*_*i *_to be small unless significant counts are observed for that gene.

**Figure 2:**
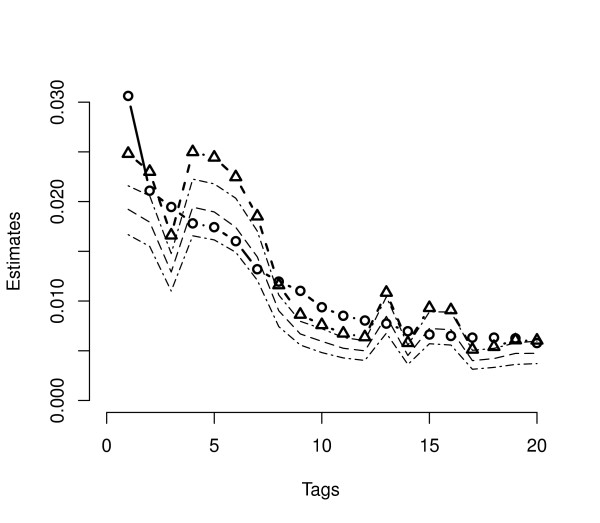
**Dirichlet-Poisson-Binomial estimates with the flat prior**. Probability estimates and inferences for *S. cerevisiae *log-phase data based on the DPB model with flat prior, *α*_*i *_= 1, for all genes. The 20 genes with the largest tag counts are arranged in decreasing rank order along the *x*-axis. The observed tag proportions are marked in open circles, the bias corrected MLE in open triangles. The analytically computed posterior mode when *α*_*i *_= 1 coincides exactly with the corrected MLE. Also included are the estimated posterior mean and upper and lower marginal 95% Bayesian posterior bounds based on MCMC sampling.

**Figure 3:**
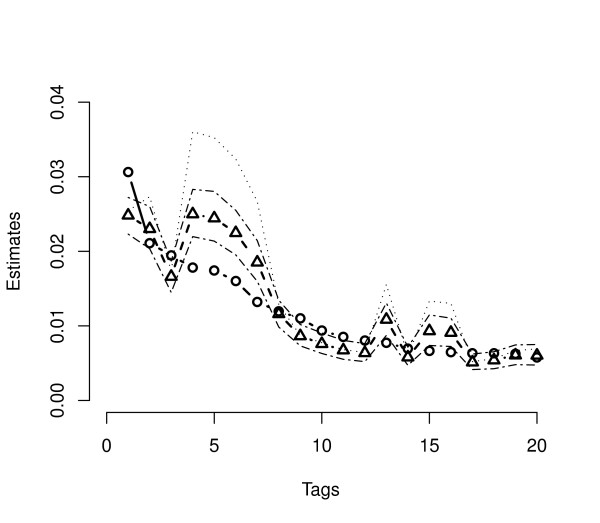
**Dirichlet-Poisson-Binomial estimates with the tub prior**. Probability estimates and inferences for *S. cerevisiae *log-phase data based on the DPB model with tub prior, *α*_*i *_= 1/*l*, for all genes. The 20 genes with the largest tag counts are arranged in decreasing rank order along the *x*-axis. The observed tag proportions are marked with open circles, the bias corrected MLE in open triangles. In this case analytically derived posterior modes deviate substantially from the corrected MLE while the estimated posterior mean is identical to it. Upper and lower marginal 95% Bayesian posterior bounds are also given.

To analyze performance, we applied each algorithm to data collected from the log-phase growth of *S. cerevisiae *and analyzed by [7]. Here, *l *= 6, 178 is the number of genes in the sample. For each of the three methods, we compared posterior means from the Gibbs sampler to unadjusted multinomial MLE's, corrected MLE's based on Eq. (4), and the analytically derived joint posterior mode given in [6]. In addition, 95% marginal posterior intervals are also computed for each gene from the Gibbs sampler output. Figure 2 presents results from DPB analysis. Both estimates and intervals generated using the *flat *prior are plotted versus rank for the 20 genes with largest tag counts. Equations (3) and (5) dictate that the corrected MLE and the posterior mode coincide perfectly in this case. Both posterior means and posterior modes based on the DPB bias correction deviate as much as 40% from the unadjusted MLE for many of the most frequently tagged genes indicating the importance of the correction. Posterior modes and means follow nearly identical trends but with the modal estimates being uniformly larger. Figure 3 summarizes results when using the *tub *prior in the DPB case. Here one can see the *i*^th^corrected MLE and posterior mean now coincide up to simulation precision (≈ 1%). As with the *flat *prior, all estimators generated using the *tub *prior deviate significantly from the unadjusted MLE's.

Both Figures 2 and 3 show that modal estimates typically lie slightly above the 97.5% bound of the simulated marginal posterior distribution. The reader might wonder why the multivariate mode and mean are so far apart. The explanation lies in the fact that posterior distribution is spread across a a large parameter space consisting of many thousands of dimensions. While the posterior distribution has a skewed density with a very slight slope, it is essentially flat over most of the parameter space. While the mode is located at the most likely point, an extreme point where only a fraction of the categories have non-zero tags, the mass spread across the rest of the space pulls the mean away from this point leading to the observed separation. This is similar to the effect of skewness in a one dimensional distribution which pulls the mean away from the mode.

Graphical displays based on the results of the DMB and MD algorithms had very similar appearances and so are not shown. However, meaningful differences in posterior means generated by the methods and across the two priors do exist and are discussed in the following section.

In order to ensure that autocorrelation did not adversely effect parameter estimates, convergence analysis was performed. Posterior samples for the proportion *m*_3_, which corresponds to the open reading frame YAL003W, were investigated for all approaches. This gene was observed in 32 of the 14,285 tags and was selected randomly among the set of genes with medium to large tag counts. Autocorrelations of both sequences were tested at various lags. Results given in Table 1 suggest that the *flat *prior may generate slightly larger correlations than the *tub *but in both cases autocorrelations are very low between samples with lags as small as 10.

**Table 1:** Autocorrelation in Gibbs samples of proportions.

	DPB		DMB		MD	
**Lag**	**(*α *= 1)**	**(*α *= 1/*l*)**	**(*α *= 1)**	**(*α *= 1/*l*)**	**(*α *= 1)**	**(*α *= 1/*l*)**

10	0.011	0.000	0.015	0.019	0.053	0.008
20	0.047	0.000	-0.025	0.013	-0.012	0.010
40	-0.001	-0.028	-0.019	0.013	-0.034	0.012
80	0.000	0.027	0.000	-0.007	0.011	-0.010

Autocorrelation estimates for three proposed algorithms across posterior samples of the mRNA proportion at the the open reading frame YAL003W in *S. cerevisiae *log-phase data.

Table 2 examines correlations between posterior samples at various lags for the population size *N *in the DPB algorithm and the number of unconverted transcripts *r *from the MD method. When using the *flat *prior, high correlation among posterior simulations of *N *exist at all tested lags leading to unreliable inferences. Interestingly, the *tub *prior leads to negligible correlations among samples at all measured lags. Using the MD method, the autocorrelation in samples of *r *decreases to 0 after 50 simulations when using the *flat *prior. Samples are uncorrelated at lags as small as 10 when using the *tub *prior.

**Table 2:** Autocorrelation in Gibbs samples of population size.

	DPB		MD	
	***N***	***N***	***r***	***r***

**Lag**	**(*α *= 1)**	**(*α *= 1/*l*)**	**(*α *= 1)**	**(*α *= 1/*l*)**

10	0.905	0.024	0.606	0.007
20	0.863	0.018	0.344	-0.012
40	0.783	-0.029	0.088	-0.034
80	0.666	0.027	-0.011	0.011

Autocorrelation estimates for mRNA population size *N *in the DPB algorithm and number of unconverted transcripts *r *in the MD algorithm.

In terms of computational efficiency, with *l *= 6, 178, 5 million samples from the DPB algorithm required 21.7 and 19.3 minutes with the *flat *and *tub *priors respectively. The DMB was slightly slower taking 30.7 and 25.2 minutes to run. Finally, the MD method completed 5 million samples in 20.2 and 19.3 minutes, respectively.

In order to avoid any effects due to autocorrelation our real data experiments stored only the last of every hundred samples for analysis. Inference for means was based upon the final 1000 of these stored samples drawn after an extensive burn-in period of 400,000 simulations. Because the vector **m **is usually the quantity of interest, far fewer samples would be needed in practice. This would significantly reduce the computation times for the algorithm.

### A Simulation Study

Although all methods produce similar trends in their estimates, quantitative differences do exist. It is also important to quantify the effects of the prior distributions on the analysis. Because the inferential quality is often more relevant in scientific investigations of relative and differential expression than point estimates, we attempt to contrast inferential quality of the three methods by examining marginal 95% posterior intervals constructed for *m*_*i *_from simulated libraries. The proportion of times the computed interval covers the true known simulation proportion *m*_*i *_is then recorded. Accuracy is assessed by how close the observed coverage percentage is to the desired level of 95%. The average interval length of the 95% posterior intervals was considered as a second criteria of inferential accuracy but no meaningful differences in average interval length were observed among the three methods.

Table 3 presents average coverage levels across a range of *m*_*i *_values for library sizes *l *= 6, 178 and *l *= 1, 000. One clear conclusion is that under the simulation protocol and the priors considered, the DPB method has the best general performance across the range of *m*_*i *_values for both priors. In contrast, the DMB and MD methods, which have very similar performance with almost identical coverage values across all categories and priors, offer poor coverage for medium and large categories under the *flat *prior. All methods provide accurate coverage under the *flat *prior for the intermediate values of *m*_*i *_(i.e. .0000167 - 0.000912) which represent the bulk of the genes used in our simulations. Below this range, all three methods have a tendency to overestimate the true *m*_*i*_. This is due to the fact that the magnitude of *α*_*i *_for the *flat *prior is on the same order of magnitude as the observed tag counts and leads to poor coverage performance. In contrast, all three methods tend to underestimate the true *m*_*i *_value in the abundant categories. This *shrinkage *effect was discussed earlier; see [9] for a detailed discussion.

**Table 3:** Simulated coverage probabilities for proposed methods.

**Proportion *m***_*i *_**Range**	Library Size	Gene Count	Prior					
			Flat *α*_*i *_= 1			Tub *α*_*i *_= 1/*k*		
			
			DPB	DMB	MD	DPB	DMB	MD
0 - 1.67 × 10^-5^	6178	1181	0.800	0.855	0.854	0.104	0.104	0.104
-	1000	25	0.825	0.834	0.834	0.107	0.108	0.107
1.67 × 10^-5 ^- 1.23 × 10^-4^	6178	3678	0.947	0.975	0.976	0.520	0.520	0.520
-	1000	209	0.9425	0.950	0.950	0.558	0.558	0.558
1.23 × 10^-4 ^- 9.12 × 10^-4^	6178	1173	0.961	0.951	0.948	0.899	0.898	0.898
-	1000	578	0.952	0.957	0.957	0.911	0.911	0.911
9.12 × 10^-4 ^- 6.74 × 10^-3^	6178	133	0.939	0.485	0.479	0.945	0.944	0.944
-	1000	165	0.950	0.950	0.945	0.934	0.934	0.934
6.74 × 10^-3 ^- 1.35 × 10^-1^	6178	13	0.809	0.009	0.005	0.953	0.951	0.951
-	1000	23	0.948	0.794	0.780	0.943	0.939	0.939

Coverage percentages for 95% posterior intervals for MCMC methods. Percentages represent the average number of intervals out of 1000 which covered the true proportion *m*_*i *_over the given range of *m*_*i *_values. Standard errors of proportions reported are below 1.5% and typically close to 1%.

**Figure 4:**
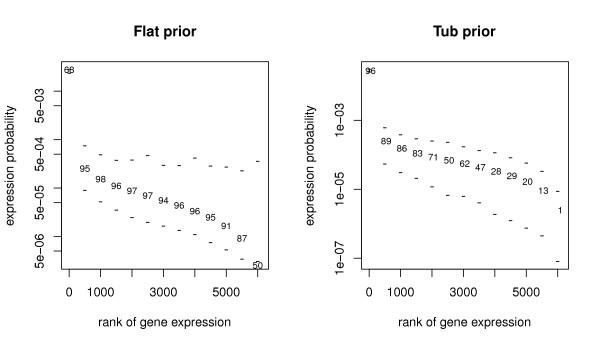
**Trends in coverage probabilities**. Comparison of marginal coverage probabilities of 95% posterior intervals for both priors across 13 genes for simulated libraries with 6,187 genes and 15,000 tags. Upper and lower bars represent the average upper and lower endpoints across 1,000 simulated libraries. Percentages shown give coverage probability. Percentages are located at the true generating proportion. The left and right panels correspond to the flat and tub priors, respectively.

**Figure 5:**
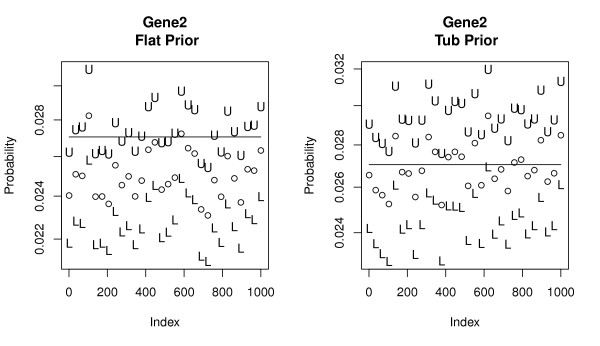
**Simulated intervals for a large proportion gene**. 95% posterior intervals for the gene with 2nd largest count across a range of simulated libraries. Left frame represents the *flat *prior (Actual Coverage = 0.652), right frame the *tub *prior (Actual Coverage = 0.961). U and L are upper and lower confidence points for each simulated value. The open circle represents the mean value of samples from each library. The solid line is the true generating proportion *m*_2 _= 0.027. *ϕ *= .959.

**Figure 6:**
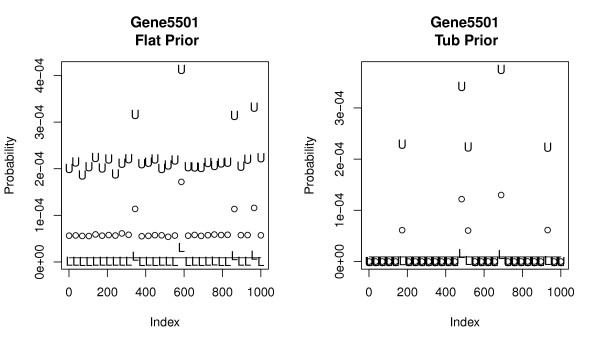
**Simulated intervals for a small proportion gene**. 95% posterior intervals for gene with 5500th largest count across a range of simulated libraries. Left frame represents the *flat *prior (Actual Coverage = 0.867), right frame the *tub *prior (Actual Coverage = 0.127). U and L are upper and lower confidence points for each simulated value. The open circle represents the mean value samples from each library. The solid line is the true generating proportion *m*_5500 _= 0.000009, *ϕ *= 0.909.

Not surprisingly, *shrinkage *effects become more pronounced as the number of genes *l *increases further degrading the quality of the estimates. This shrinkage is most acute for DMB and MD methods applied to larger library sizes, which perform poorly for the segments with the. As noted above, the *flat *prior works well across a wide range of values but performs poorly in the extremes. Figure 4 displays this effect for a subset of 13 genes. Alternatively the *tub *prior works well for the majority of genes where the *flat *prior fails. For example, the *tub *works well in large *m*_*i *_categories because it shrinks the estimates very little. Its weakness; it adds almost no mass to categories with small positive values of *m*_*i *_and, if no counts are observed, intervals are produced with both endpoints near 0. Figures 5 and 6 provide detailed views from a sample of 30 simulated libraries for a large and small *m*_*i *_gene. Focusing on the left panel of Figure 5, the shrinkage effect is evidenced by the fact that posterior mean estimates of *m*_*i *_systematically underestimate its true value. This leads to upper interval endpoints being systematically too small. The *tub *prior on the right corrects this phenomenon well. On the left of Figure 6 we see posterior mean estimates of *m*_*i *_being drawn well above their actual values by the reversing of the shrinkage for small *m*_*i *_categories. This leads to reasonably accurate coverage. In the right panel we see that *tub *prior fails miserably in this case. Intervals for genes with 0 counts collapse close to zero systematically underestimating the true *m*_*i *_values.

Because these methods are very efficient to compute, both methods can be applied to analyze a single library with the *tub *prior being relied upon for larger count categories while the *flat *prior is used for the remaining categories.

### Differential Expression in SAGE between Libraries

Tag based transcriptome sequencing such as SAGE is most commonly used to identify differential expression of genes across groups of libraries. It is important to point out the consequences of differences in tag formation probabilities in such studies along with the advantages of the proposed methods in this context.

Because the anchoring enzyme AE cutting probability *p *is experiment dependent [6], it is sensible for the tag formation mechanism to be taken into account when evaluating differential expression across experiments. Indeed, this should lead to increased power and accuracy in such studies. To briefly explore the consequences of variation in cutting probability, consider two experiments **A **and **B**. Letting *p*_*a *_and *m*_*ia *_represent the cutting probability and relative proportion of mRNA for the *i*^th ^tag in experiment **A **and similarly for **B**, the theoretical odds ratio for the tag proportions across libraries **A **and **B **is(6)

where  is the tag formation probability for tag *j *in experiment **A**, Ω is the true odds ratio, and *k*_*j *_is the number of AE positions upstream from which the tag was derived. The approximation holds when both *p*_*a *_and *p*_*b *_are large (i.e. > 0.5) and the *i*^th ^expression level *m*_*i *_is small in an absolute sense for both experiments, conditions that are almost universally met in SAGE studies.

If only data derived from the 3' most tagging site of each gene is analyzed so that *k *= 0, and *ϕ*_*ja *_= *p*_*a *_then the odds simply reduces to (*m*_*ia*_(1 - *m*_*ib*_))/(*m*_*ib*_(1 - *m*_*ia*_)), the exact odds ratio desired. However, suppose all tags are allowed and the experimenter tests differential expression for the *i*^th ^tag, which derives from an AE site 1 position upstream from the 3' end. The tag formation probability now takes the form *p*(1 - *p*). Assuming cutting probabilities *p*_*a *_= .92 and *p*_*b *_= .96 we see that our estimate of the odds ratio is overestimated by a factor of ~1.9 potentially leading to a false positive diagnosis. In fact, this phenomenon could result in a sizeable proportion of both false positive and false negative conclusions. If SAGE analysis were restricted to 3' most AE sites and all data from upstream tags were eliminated from the library, *ϕ*_*i *_would be constant and sampling bias would be eliminated by analysis using odds ratios (logistic regression coefficients are estimated log-odds ratios). Once again, the caveat to this approach is that a significant proportion of the observed tags may be discarded. The need for the adjustments we propose stems from an effort to accurately combine multiple tags arising from the same mRNA and offers the ability to utilize a much larger fraction of the data collected leading to more power for differential testing.

Monte-Carlo sampling mechanisms such as DPB can easily be applied to evaluate differential expression across groups of libraries. In the case of comparison for gene *i *across two libraries, individual Monte Carlo samples from each library can be differenced, *m*_*i*1 _- *m*_*i*2_, and the distribution of these differences used for inference; see [[9], Section 6.]. As in a paired t-test, if the bulk of differences fall far from 0 we may conclude that differential expression exists. Alternatively, it may be more appropriate to form odds ratios based on the sampled quantities and see if the bulk of these are far from 1.

The Monte-Carlo sampling nature of the algorithms presented here and their computational efficiency leads to several advantages over recent methods [14-16]. First, and foremost the ability to use a larger fraction of the data collected while simultaneously correcting bias is critical to improving power. In addition, MCMC samples allow both marginal and joint testing for differential expression across all genes using a single sampling run for each library involved. Joint testing of differential expression is possible because posterior samples represent the joint posterior distribution of the proportions **m**. Importantly, our method requires no asymptotic conditions to be met in order to guarantee the validity of the results.

## Conclusions

This work focuses on a general biasing mechanism that may have a significant impact on the interpretation of libraries generated by SAGE and closely related methods. Beyond drawing attention to the consequences of incomplete digestion, two main contributions are presented. First, we derive and analyze three Bayesian algorithms that provide corrected posterior inference for relative proportions of genes or tags in the overall population. Second, through calculations we deduce the consequences of tag formation bias on testing of differential expression and discuss how the algorithms derived here can be used to correctly assess differential expression.

This work compliments the earlier work in [6] by providing a flexible and efficient methodology which extends the range of the prior distributions that are available. It also allows researchers access to interval estimates in order to assess uncertainty in the estimated quantities.

The proposed methods are appropriate when incomplete digestion is the main source of multiple tag generation. They may be applied to both tag level data and to inference at the gene level based on aggregated counts. If possible, aggregating tags will provide better inferences due to larger available counts. Of the three approaches, the DPB method provided the most accurate confidence intervals over the widest range of relative proportions and we recommend its exclusive use. In addition, the choice of prior also plays an important role in the effectiveness of the algorithm. Of the two priors tested, the *flat *prior was effective over a wide range of categories but resulted in excessive shrinkage for the most abundant categories. For a fixed number of sampled tags, this shrinkage effect becomes more severe as one increases the number of categories. Hence, aggregating tags provides a second advantage since it greatly reduces the number of categories used in an analysis. The proposed *tub *prior provides little shrinkage and posterior intervals based on this prior are very accurate for abundant categories. In practice, both priors should be applied and inference should be based on the samples with the larger average.

We note that the sampling algorithms presented are independent of the model for tag formation probability and models other than the geometric model discussed can easily be substituted. It should also be possible to extend the proposed methods using a mixture methodology as suggested by [9].

Correction of tag formation bias may play a significant role in improving power in differential expression studies based on SAGE or related tag libraries. To ensure valid testing, one must either include bias correction or eliminate non-3' tag counts from the analysis. Furthermore, the ability to effectively test for changes in groups of genes (gene networks) is now possible due to the multivariate nature of the posterior distribution. This advantage may become significantly more important as more elaborate studies and techniques are developed in the future.

Due to the overall simplicity of genome architecture, alternative splicing is generally not a problem when analyzing gene expression in *S. cerevisiae *and other microorganisms. However, alternative splicing is widespread in most multicellular organisms [1,2] and greatly complicates transcriptome analysis. While the adjustments we propose do not solve the interpretation problems caused by alternative splicing, properly identifying multiple tags from common mRNA is a necessary first step in doing so. A logical next step would be to expand the tag formation model underlying this work by allowing a single gene to produce multiple mRNA isoforms instead of a single type. One approach would be to extend our model in a manner similar to the approach developed for analyzing mRNA sequence data developed by [20].

Extending our model in such a way should allow us to make inferences about the distribution of mRNA isoforms produced by a gene based on the distribution of tags experimentally observed.

Conceptually, the problems caused by alternative splicing are similar to those caused by tag ambiguity between different genes. For example, in the case of alternative splicing, the challenge is in determining the mRNA isoform from which each tag originates. Similarly, in the case of ambiguous tags, the challenge is in determining the gene from which each tag originates. Thus it is plausible that data interpretation problems caused by both alternative splicing and ambiguous tags could be overcome by extending our model to include multiple sources of a single tag.

## Methods

### The Data

We explicitly consider three strategies to simulate from the posterior distribution given by Eq. (5). The algorithms discussed are tested for inferential accuracy using simulated data as well as being applied to a published SAGE data set that analyzes a yeast organism *S. cerevisiae *[7]. The published data was collected from the log-phase. There are 6,178 genes included in the data. The maximum tag count was 392 and the minimum was zero. 3,560 of the genes included had observed counts of 0. Gene dependent sampling probabilities range from ≈ .3% to 100%. Tag assignments to unique genes and assignment of non-unique tags for this analysis were described in [6].

### Computing and Software

All simulations described below were computed using R-environment (Version 2.6.2) on an 8 core, Intel Xeon 2.66 GHz desktop server running Linux Ubuntu 8.04. R-scripts that implement the Gibbs sampling algorithms described below are provided; see Additional file 1.

### The Dirichlet-Poisson-Binomial (DPB) Model

The DPB approach, inspired by Casella and George [21], assumes a fixed population of mRNA's of size *N *exists within the sampled cells. The total size *N *is random across samples and follows a gamma distribution that is rounded down to the nearest integer. The choice of gamma here is convenient due to its role as a conjugate prior to the Poisson distribution. Given *N*, the relative proportions of the categories of mRNA, *m*_*i*_, are unknown and assumed to follow a Dirichlet distribution with prior  = (*α*_1_, ..., *α*_*l*_). The *α*_*i *_will typically be identical resulting in objective inference.

Because cells contain mRNA transcripts from thousands of different genes, the probability of seeing any particular type is low. It is therefore logical to assume, given *N *and *m*_*i*_, that the actual number of mRNA's of a certain type *g*_*i*_, *i *= 1, ... *l*, extracted from the group of cells, satisfies a Poisson distribution with mean *μ *= *N*·*m*_*i*_. Finally, the restriction enzyme process generates a tag count *t*_*i *_for a particular mRNA transcript in a binomial fashion based upon the tag formation probability *ϕ*_*i*_. Hence, the hierarchical data generating mechanism follows, *T*_*i*_~*Binomial*(*g*_*i*_, *ϕ*_*i*_), *g*_*i*_~*Poisson*(N * *m*_*i*_), **m **~*D*_*l*_(), and *N ~ceiling*(*Gamma*(*γ*_1_, *γ*_2_)). We refer to this model as the Dirichlet-Poisson-Binomial (DPB) model.

A first weakness of this model is that the sum of all counts ∑*g*_*i *_may not add up to the total counts *N*. However, this approach allows us to infer the total population size *N*. Together, the elements described above give a joint posterior distribution,

¿From this expression, we can deduce the set of full conditional distributions,

where *δ *= (, *γ*_1_, *γ*_2_). Gibbs sampling can be implemented by iteratively simulating from each conditional after replacing any unknown random quantities in the conditioning set with simulation values from the previous iteration; see [22].

The hierarchical model considered here is not identical to Eq. (3), but depends upon prior parameters (*γ*_1_, *γ*_2_) which effect the mode of the posterior distribution. One possibility for choosing values of these parameters is to minimize the distance between the approximate analytical mode discussed in [6] and the mode of this model which can be computed through iterative maximization of the conditionals; see [23,24] for further details.

In our simulations we used a shape parameter *γ*_1 _= 100 and prior scale *γ*_2 _= 200. The mean of a gamma with these parameters is 20,000 which is somewhat larger than a natural estimate of the population size, ∑_*i*_(*t*_*i*_/*ϕ*_*i*_) = 16438.81. This was chosen intentionally to determine if the posterior would converge to a reasonable estimate of *N*.

### The Dirichlet-Multinomial-Binomial (DMB) Model

The Dirichlet-Multinomial-Binomial (DMB) approach is arguably more faithful to the sampling mechanism inherent in SAGE than DPB. *N*, the total number of mRNA in the sample is assumed to follow a Poisson distribution with mean *λ*. Given this mRNA population size, the vector **g **of counts for each category of mRNA prior to tag formation follows a multinomial distribution,

whose probabilities are the mRNA proportions *m*_*i *_and are assumed to follow a Dirichlet distribution with parameter vector . Reminding readers that  = (*ϕ*_1_, *ϕ*_2_, ..., *ϕ*_*l*_) is the vector of tag formation probabilities and letting *δ *= (*λ*, *γ*_1_, *γ*_2_), the full conditional distributions are derived to be,

where m *(1 - ) is an element-wise product with 1 representing a vector of identical dimension to **m**. Hence, the *i*^th ^element of the vector is given by *m*_*i *_(1 - *ϕ*_*i*_).

This formulation is more natural in the sense that it restricts the pre-tagging counts to sum to the population total *N*. One drawback is that the observed data provides no information for posterior inference about the population size *N*. Iterative maximization for mode calculation is not viable due to the discrete nature of the multinomial conditional distribution.

### The Missing Data (MD) Model

Instead of re-normalizing the probabilities *θ*_*i *_= (*m*_*i*_*ϕ*_*i*_)/(∑*m*_*i*_*ϕ*_*i*_) and basing inference on the posterior of Eq. (5), a more straightforward approach uses the concept of missing data which is often associated with the EM algorithm; see [22]. We augment the observed data vector **t **with an extra category that represents any cDNA that is not converted to a tag. This count, *r*, is not observed, but if a distribution such as the Poisson is proposed, we may consider a Bayesian approach. Given the value of *r *and assuming a Dirichlet prior on the unknown initial proportions **m **~D_*l*_(*α*_1_, ..., *α*_*l*_), the resulting posterior distribution for **m **is,

This resulting posterior distribution is a Dirichlet distribution with *l *+ 1 categories compared with the *l *categories in the prior distribution. Exact computation of the conditional expectations *E*(*m*_*i*_|*r*) is now straightforward. Using the formula for the mean of a Dirichlet distribution

Summing the expectation over *m*_*i*_, which must sum to one, gives  which is intuitively reasonable. Substituting this into the previous expression eliminates the dependence upon *r*. Hence, the marginal expectation of *m*_*i *_across values of *r *is(7)

Gibbs sampling is also available for this problem and extends inference to Bayesian interval estimates. If *r ~Poisson*(*μ*) then the posterior distribution can be written,

The full conditional distributions are

In order to apply the Gibbs sampler a value for the prior mean of *r*, *μ *must be selected. A logical mean for the Poisson is *t*_*tot *_∑_*i*_*m*_*i*_(1 - *ϕ*_*i*_)/∑_*i*_*m*_*i *_*ϕ*_*i *_since ∑*m*_*i*_(1 - *ϕ*_*i*_) = 1 - ∑*m*_*i *_*ϕ*_*i *_is the probability that an mRNA is not converted into a tag. Equation (4) provides a useful estimate of ∑*m*_*i *_*ϕ*_*i *_in the case of a *flat *prior.

Like the DPB algorithm above, the missing data algorithm also admits an iterative optimization procedure in order to compute the posterior mode. The mode of the Dirichlet conditional given by [22] is  = (*t*_*i *_+ *α*_*i *_- 1)/(∑_*i*_(*α*_*i *_+ *t*_*i*_) + *r *- *l*) while the mode for the Poisson is the mean rounded down to the nearest smaller integer. For example, considering a *flat *prior for **m **along with a prior mean of *μ *= 10, 100 for *r*, the posterior mode of the missing data model is nearly identical to the exact analytical mode given in [6]. It is tempting to invoke the argument used to derive Eq. (7) to obtain the marginal mode.

Unfortunately, when *α*_*i *_< 1 and *t*_*i *_= 0 this leads to estimates outside of the parameter space.

### Simulation Study Protocol

Simulation of libraries was based upon the proportions estimated from the aggregated *S. cerevisiae *log-phase data described earlier. First, long and short multinomial proportion vectors **m **of lengths *l *= 6, 178 and 1, 000 respectively were constructed. Actual mean estimates from the log-phase library were adopted for the case *l *= 6, 178 while for *l *= 1, 000, **m **was based on renormalizing the first 1,000 elements of the longer case. The vector of known tag formation probabilities, {*ϕ*_1_, ..., *ϕ*_*l*_} was constructed by simulating a theoretical number of AE cleaving sites and then using Eq. (1) to compute individual probabilities *ϕ*_*i*_. The number of cleaving sites followed a Poisson distribution with mean 2 with 1 count added to each category. This ensured that each "gene" has at least one cleaving site. The cleavage probability was set at *p *= .55 based on estimates given in [6].

Using the above protocol, for each combination of library size, *l*, and prior, 1,000 libraries, **t**_*j*_, each containing *n *= 15, 000 tags were simulated. For each simulated library, each of the three proposed algorithms was used to generate 20,500 posterior sample vectors **m**_*jk*_, *k *∈ 1, ...20, 500. Every 20th sample was collected after a burn in period of 500 samples resulting 1,000 Monte Carlo samples of **m **for each method. Samples were then used to generate mean, median and 95% marginal posterior intervals for each of the *l *gene categories. Coverage percentages for the *i*^th ^gene category are computed by finding the proportion of times that the interval for category *i *from a particular method contains the true proportion *m*_*i *_across the 1,000 simulated libraries **t**_*j *_. Figure 4 displays a sample of coverage percentages across a range of categories for the DPB method under two different prior values.

## Authors' contributions

RLZ devised and implemented the algorithms, computed the experiments and wrote the majority of the manuscript. MAG developed the tag formation model, prepared the real data, wrote parts of the manuscript, and provided subject matter expertise and computational resources. WMB helped edit the manuscript and construct graphical displays. AA assisted in development of the algorithms. All authors have read and approved this manuscript.

## Supplementary Material

Additional file 1 **SAGEGibbs.R**. File contains a script which implement the Gibbs Samplers described in the paper. The script is written in the R-environment. Click here for file
